# The Effect of Vitamin D Deficiency on Immune-Related Hub Genes: A Network Analysis Associated With Type 1 Diabetes

**DOI:** 10.7759/cureus.68611

**Published:** 2024-09-04

**Authors:** Safin Hussein, Fatemeh Bandarian, Najmeh Salehi, Ali Mosadegh Khah, Elahe Motevaseli, Zahra Azizi

**Affiliations:** 1 Molecular Medicine, School of Advanced Technologies in Medicine, Tehran University of Medical Sciences, Tehran, IRN; 2 Biology, College of Science, University of Raparin, Ranya, IRQ; 3 Endocrinology and Metabolism Research Center, Endocrinology and Metabolism Clinical Sciences Institute, Tehran University of Medical Sciences, Tehran, IRN; 4 School of Biology, College of Science, University of Tehran, Tehran, IRN; 5 Endocrinology, AJA University of Medical Science, Tehran, IRN

**Keywords:** protein-protein interaction (ppi) network, hub genes, differentially expressed genes, immune-relevant genes, type 1 diabetes (t1d), vitamin-d, bioinformatics analysis

## Abstract

Background

Type 1 diabetes (T1D) is an autoimmune disorder that results in the destruction of pancreatic beta cells, causing a shortage of insulin secretion. The development of T1D is influenced by both genetic predisposition and environmental factors, such as vitamin D. This vitamin is known for its ability to regulate the immune system and has been associated with a decreased risk of T1D. However, the specific ways in which vitamin D affects immune regulation and the preservation of beta cells in T1D are not yet fully understood. Gaining a better understanding of these interactions is essential for identifying potential targets for preventing and treating T1D.

Methods

The analysis focused on two Gene Expression Omnibus (GEO) datasets, namely, GSE55098 and GSE50012, to detect differentially expressed genes (DEGs). Enrichr (Ma'ayan Laboratory, New York, NY) was used to perform enrichment analysis for the Gene Ontology (GO) biological process and Kyoto Encyclopedia of Genes and Genomes (KEGG) pathways. The Search Tool for the Retrieval of Interacting Genes 12.0 (STRING) database was used to generate a protein-protein interaction (PPI) network. The Cytoscape 3.10.1 (Cytoscape Team, San Diego, CA) was used to analyze the PPI network and discover the hub genes.

Results

The DEGs in both datasets were identified using the GEO2R tool, with a particular focus on genes exhibiting contrasting regulations. Enrichment analysis unveiled the participation of these oppositely regulated DEGs in processes relevant to the immune system. Cytoscape analysis of the PPI network revealed five hub genes, MNDA, LILRB2, FPR2, HCK, and FCGR2A, suggesting their potential role in the pathogenesis of T1D and the response to vitamin D.

Conclusion

The study elucidates the complex interaction between vitamin D metabolism and immune regulation in T1D. The identified hub genes provide important knowledge on the molecular pathways that underlie T1D and have the potential to be targeted for therapeutic intervention. This research underscores the importance of vitamin D in the immune system's modulation and its impact on T1D development.

## Introduction

Type 1 diabetes (T1D) represents a significant clinical condition marked by the autoimmune attack of pancreatic beta cells, leading to an insufficiency of insulin secretion. Patients are consequently incapable of effectively regulating their blood glucose levels. T1D affects around 5 to 10% of children globally [1]. T1D has detrimental effects on various body systems, including renal, ocular, cardiovascular, autonomic, and peripheral nervous systems [2]. In addition to insulin therapy, the treatment for T1D involves a focus on both protecting the remaining β-cells and promoting their regeneration [3]. Still, little is known about the pathophysiology of this multifactorial disease. The cause of T1D is a complex combination of genetic susceptibility and environmental risk factors. There is frequently a significant family history, suggesting a genetic factor, although genetics alone does not entirely account for the onset of the illness. This is because the risk of developing T1D is around ten times higher in first-degree relatives of T1D patients compared to the general population [4]. Scientists are actively studying the intricate relationship between the genes that make individuals more vulnerable and the impact of environmental risk factors. Among these risk factors, vitamin D deficiency has been identified as a key element that modulates inflammatory immune responses, ultimately resulting in the gradual loss of beta cells [5]. Understanding how certain genes are differentially expressed in response to such factors is crucial to unraveling the molecular mechanisms driving beta cell destruction in T1D.

Vitamin D has been identified as a potential environmental element that contributes to developing autoimmune illnesses, such as T1D [6]. Vitamin D is distinctive in functioning as both a vitamin and prohormone within the human body [7]. Certain genes are crucial in facilitating transportation, metabolism, and cellular reception [8]. Beyond its classical role in bone health, vitamin D has been recognized for its extensive immunomodulatory effects affecting immune cell behavior and inflammation [9]. It has been demonstrated that vitamin D can modulate the expression of various immune-relevant genes, impacting both innate and adaptive immune responses [10]. These effects often manifest as differentially expressed genes (DEGs) that influence inflammation, immune regulation, and beta-cell function, contributing to the progression or protection against T1D [11]. Population-based research has revealed a correlation between elevated levels of vitamin D in the blood and a decreased likelihood of developing T1D [12]. Current research endeavors to elucidate the molecular processes by which vitamin D interacts with genetic risk factors, increasing vulnerability to autoimmune disorders such as T1D. Disruptions in genetic pathways associated with the metabolism of vitamin D may contribute to the development of islet inflammation and autoimmunity due to vitamin D's capacity to modulate the immune system and regulate inflammatory responses [5]. Studying the complex connections between genes that affect vitamin D physiology and immunoregulation is crucial for comprehending the development of T1D and discovering novel approaches for its prevention or treatment.

Bioinformatics approaches can pinpoint patterns of genetic variation or differences in gene expression levels associated with autoimmune activation. The Gene Expression Omnibus (GEO), a pivotal resource in bioinformatics, is a repository for an extensive array of gene expression profiles across various diseases [13]. Its significance lies in facilitating a comprehensive analysis of gene expression patterns, offering a window into the molecular underpinnings of numerous pathologies, including T1D [14]. By mining this database, researchers can uncover novel genetic insights. This includes the identification of hub genes, which are key regulatory genes that play a central role in gene interaction networks and may drive disease processes [15]. A deeper understanding of gene expression in vitamin D-deficient T1D patients is critical. Vitamin D deficiency has been linked with the onset and progression of T1D, and its role in modulating the expression of genes involved in immune regulation and beta-cell function is a subject of growing research [16]. Thus, studying the gene expression patterns and identifying hub genes within the context of vitamin D metabolism is essential for unraveling the molecular mechanisms underlying T1D. Using these bioinformatics tools allows researchers to identify important hub genes that may be affected by both T1D and vitamin D levels, providing new targets for intervention [17].

By identifying key immunomodulatory genes affected by vitamin D in T1D, this investigation has the potential to offer fresh insights into the onset and pathogenesis of T1D, paving the way for novel therapeutic approaches that target these genetic pathways. Therefore, the aim of this work was to identify the hub genes affected by T1D and vitamin D status.

## Materials and methods

Approach to hub gene identification

**Figure 1:**
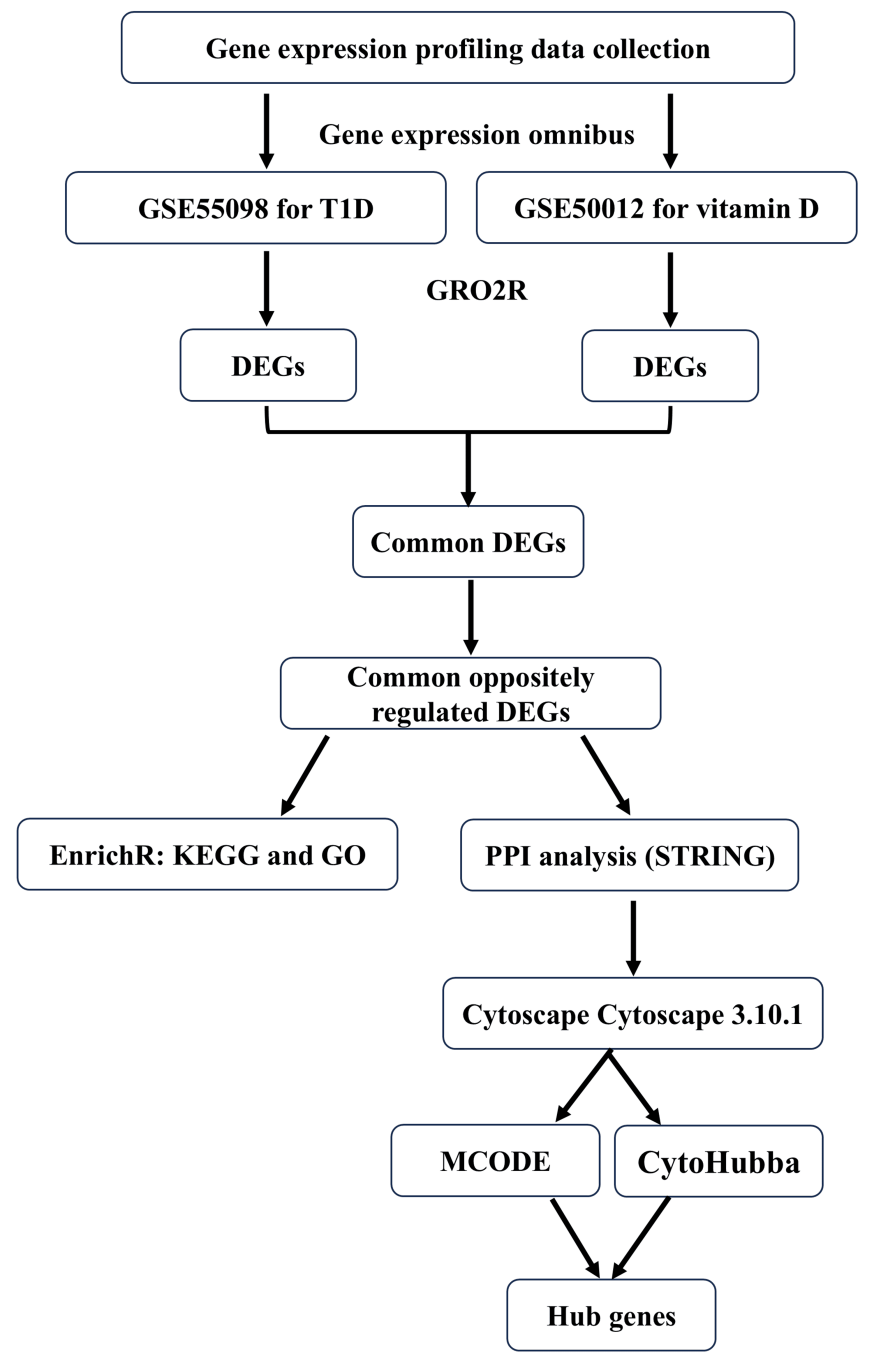
Flowchart of hub genes identification. DEGs: differentially expressed genes, GO: Gene Ontology, KEGG: Kyoto Encyclopedia of Genes and Genomes, MCODE: Molecular Complex Detection, PPI: protein-protein interaction, STRING: Search Tool for the Retrieval of Interacting Genes, T1D: type 1 diabetes

The study followed a systematic workflow to identify key immunomodulatory genes associated with T1D and vitamin D metabolism (Figure 1). First, gene expression data were searched in the GEO database. The GSE55098 dataset was used for T1D, and the GSE50012 dataset was used to study the effect of vitamin D on the gene expression of peripheral blood mononuclear cells. GEO2R, a web tool used to compare groups of samples in a GEO Series and identify DEGs across experimental conditions, was used to identify DEGs in both datasets of interest. The DEGs from both datasets were then compared to identify common DEGs, with particular attention given to those that were oppositely regulated. These common oppositely regulated DEGs were further analyzed using Enrichr (Ma'ayan Laboratory, New York, NY) to explore relevant Kyoto Encyclopedia of Genes and Genomes (KEGG) pathways and Gene Ontology (GO) terms [18]. Additionally, a protein-protein interaction (PPI) network was constructed using the Search Tool for the Retrieval of Interacting Genes 12.0 (STRING) database [19]. Key hub genes within this network were identified using Cytoscape 3.10.1 (Cytoscape Team, San Diego, CA), specifically by utilizing the Molecular Complex Detection (MCODE) and CytoHubba plugins.

Gene expression profile data

We obtained the profiling data of gene expression from the online GEO database (http://www.ncbi.nlm.nih.gov/geo/, Accessed April 11, 2024). The GSE55098, based on the GPL570 platform, was composed of newly diagnosed 12 T1D samples and 10 normal control samples. On the other hand, the vitamin D dataset GSE50012 was based on GPL10558, which had 72 samples. However, 12 peripheral blood mononuclear cells treated with vitamin D for 24 hours, compared to 12 control samples, were included in the analysis.

Microarray data

The GEO2R tool was utilized to identify DEGs in both datasets. GEO2R encompasses a wide range of experimental data forms and concepts. Gene expression fold-change (FC) was determined using a threshold criterion of |log2FC| ≥ 0.5. For both GSE55098 and GSE50012, the p-values were set at <0.05. The volcano plot was generated using GEO2R, displaying all the significant DEGs. The Venn diagram was created using the VennDiagram (Hanbo Chen, Fred Hutchinson Cancer Research Center, Seattle, WA) tool in the R language to visually display the number of DEGs in each dataset and the DEGs common to both datasets.

Enrichment analysis of DEGs

The KEGG pathway and GO analyses, including biological process (BP) enrichment of common oppositely regulated DEGs, were performed using the internet-based platform Enrichr (https://maayanlab.cloud/Enrichr/ Accessed 20 April 2024). An adjusted p-value < 0.05 was considered to be statistically significant.

PPI network construction

The PPI network was obtained from STRING 12.0 (https://string-db.org/, Accessed April 25, 2024) using default parameters and visualized in Cytoscape v3.10.1. The significance was based on a required score of >0.4, the medium confidence level. The MCODE and CytoHubba plugins of Cytoscape were used to select the hub genes. The MCODE application was used to examine modules in the PPI network. A cutoff threshold of MCODE score > 3 was established using the default parameters (degree cutoff ≥2, node density cutoff ≥ 0.1, node score cutoff ≥ 2, K-core ≥ 2, and maximum depth = 100). Ultimately, the CytoHubba plugin was employed to identify the hub genes with the highest maximum clique centrality (MCC) score [20].

## Results

Identification of DEGs

**Figure 2:**
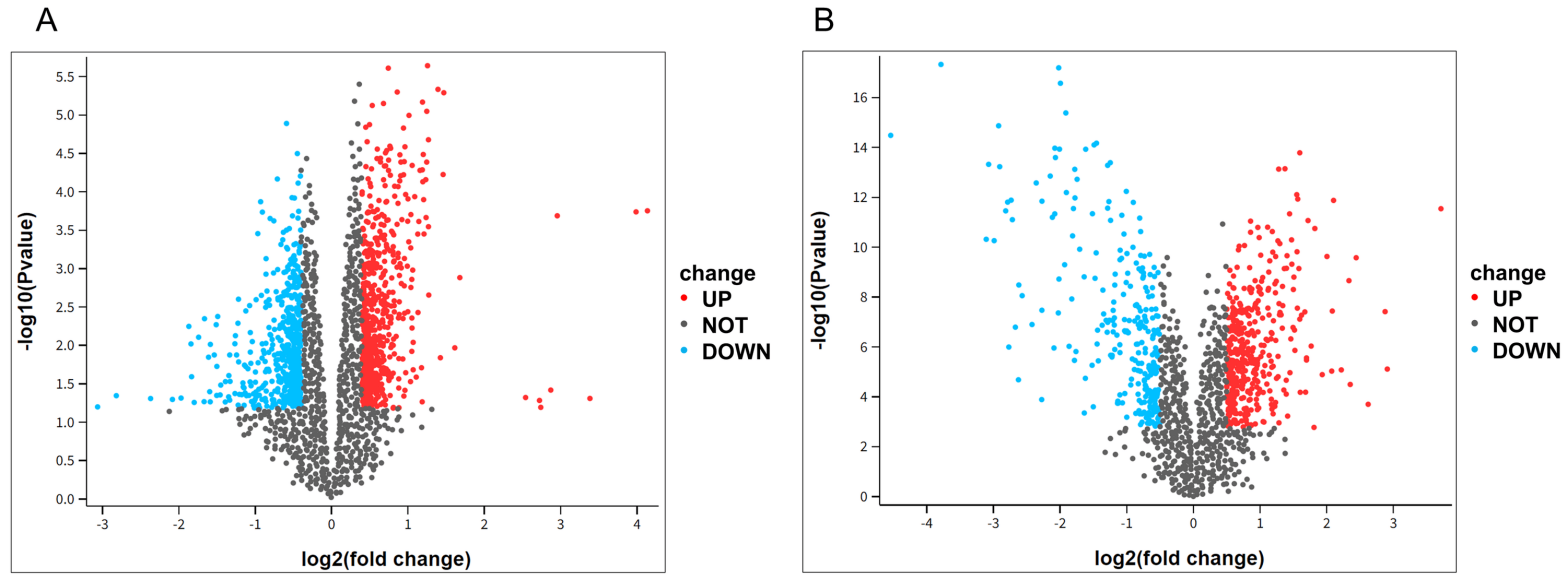
DEGs in gene expression profiles. (A) Represents the DEGs of GSE55098 between recently diagnosed T1D and normal controls. (B) Illustrates the DEGs of GSE50012 between vitamin D treatment and control samples. DEGs: differentially expressed genes, T1D: type 1 diabetes

We analyzed two microarray datasets from two separate studies. The goal was to identify DEGs specifically dysregulated in recently diagnosed T1D (GSE55098) and Vitamin D treatment (GSE50012). Genes that showed differential expression in each dataset are presented by a volcano plot (Figure 2).

**Figure 3:**
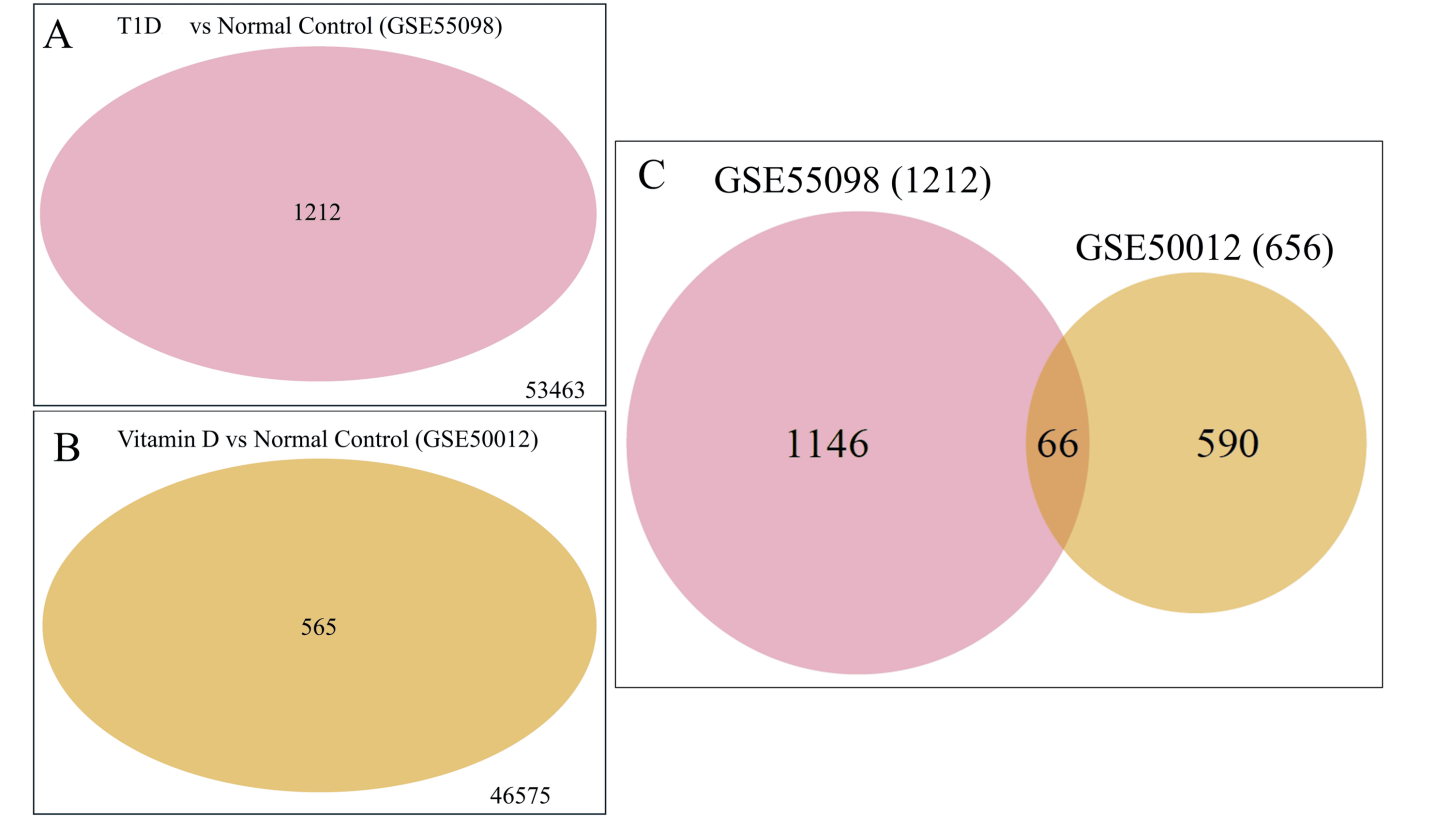
Venn diagram of DEGs in the two datasets. (A) Represents DEGs of GSE55098 between newly diagnosed T1D and normal controls. (B) Shows DEGs of GSE50012 between vitamin D treatment and control samples. (C) Summarizes the total and overlapped DEGs between the two expression profiles. DEGs: differentially expressed genes, T1D: type 1 diabetes

A total of 1,212 DEGs were identified in GSE55098, and 656 DEGs were detected in GSE50012. The two datasets shared 66 DEGs (Figure 3), 42 of which exhibited opposing regulation.

Enrichment analyses of overlapped DEGs

**Figure 4:**
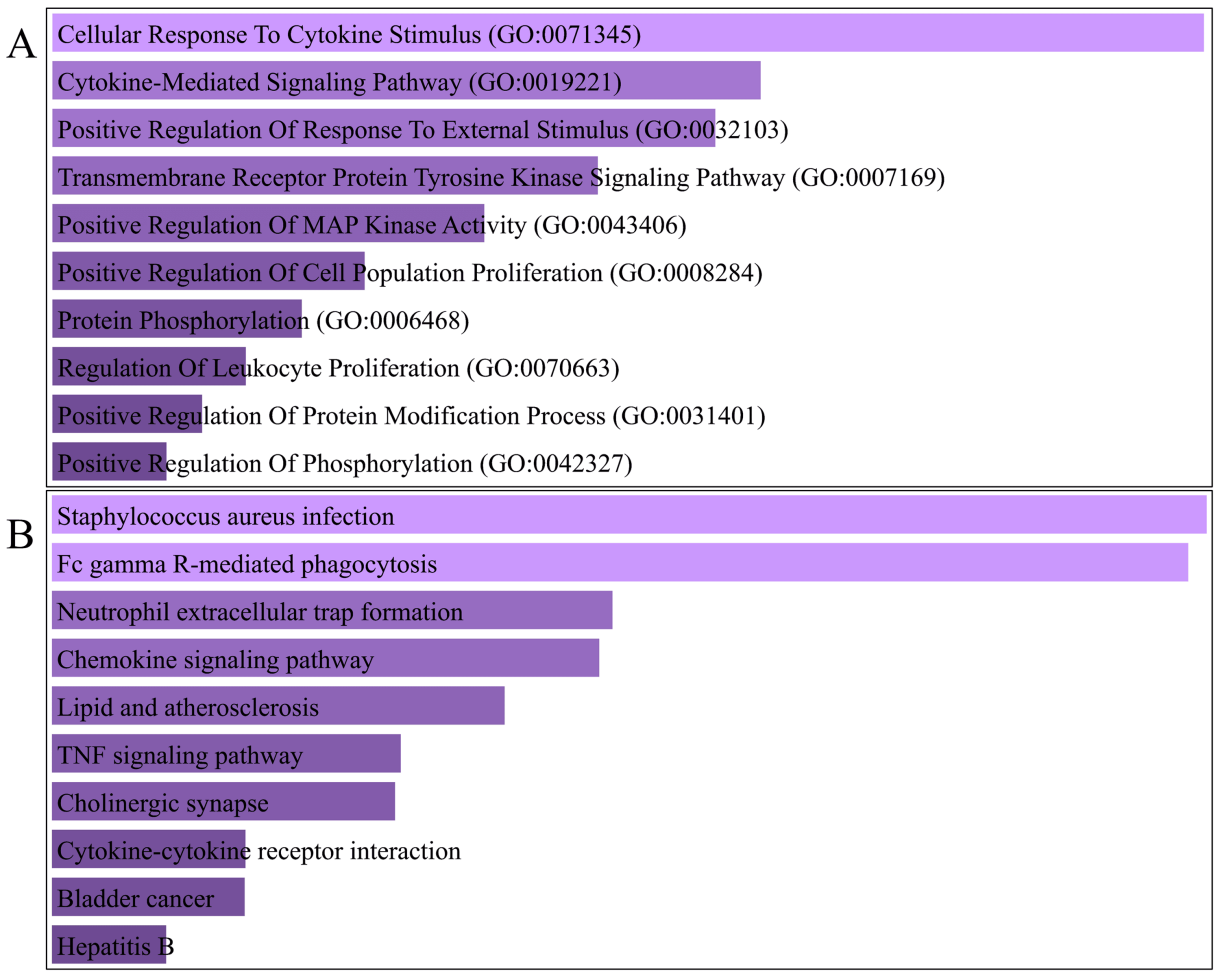
Top 10 significant Enrichr BP 2023 ontologies (A) and KEGG 2021 pathways (B) of oppositely regulated DEGs. Terms are sorted by p-value ranking. BP: biological process, DEGs: differentially expressed genes, GO: Gene Ontology, KEGG: Kyoto Encyclopedia of Genes and Genomes

According to GO analysis, the 42 overlapping genes with opposite regulation are engaged in multiple immune-related BPs, including cellular response to cytokine stimulus, cytokine-mediated signaling pathway, and positive regulation of response to external stimulus. Furthermore, the DEGs exhibited enrichment in various KEGG pathways related to the immune system, such as *Staphylococcus aureus* infection, Fc gamma R-mediated phagocytosis, and neutrophil extracellular trap formation. The findings of the enrichment analysis indicated 95 BP keywords and nine KEGG pathways of DEGs (adjusted p-value < 0.05). Figure 4 displays the top 10 items for each analysis.

PPI network and hub gene selection

**Figure 5:**
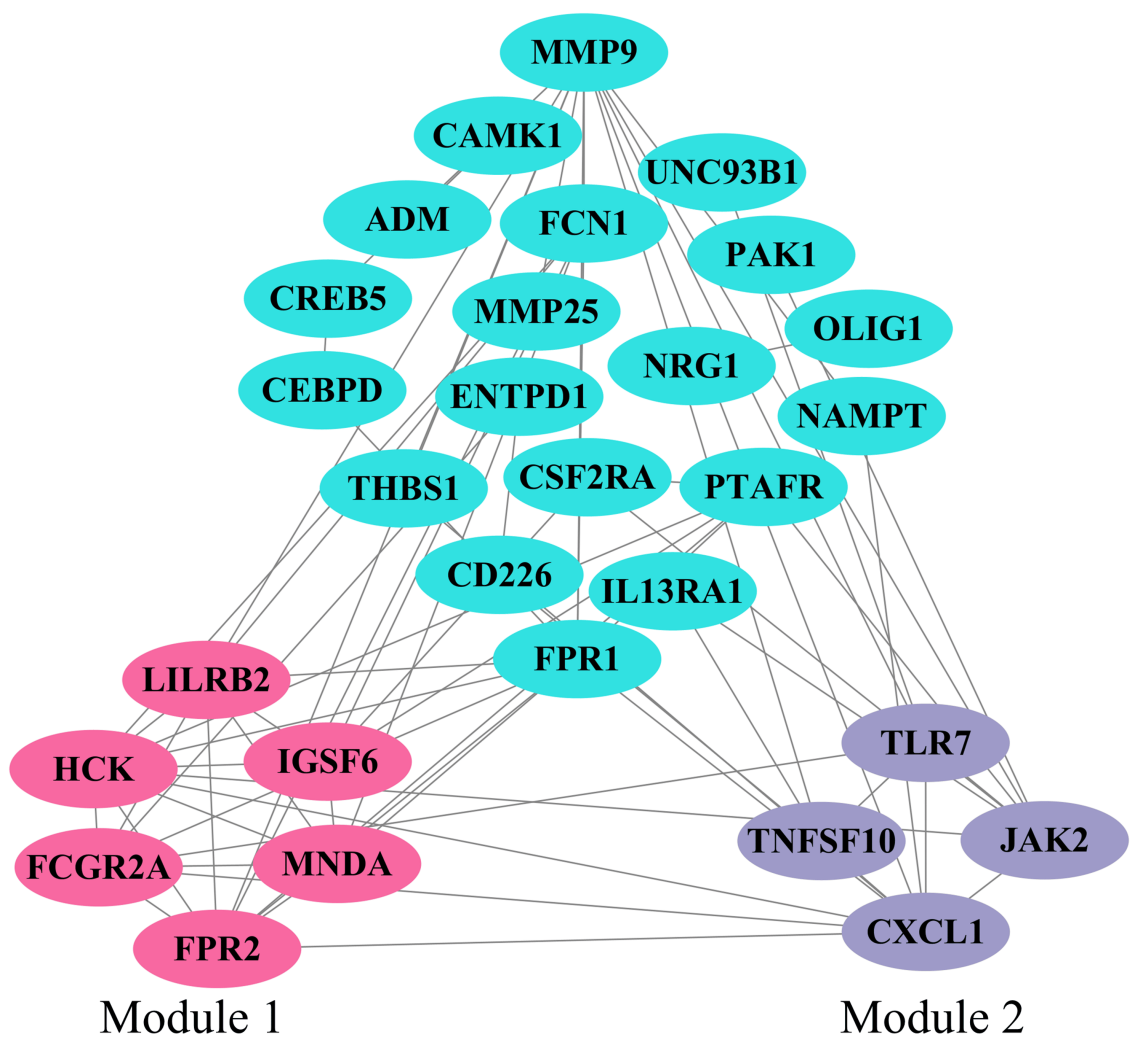
Analysis of PPI networks. The turquoise nodes represent the DEGs. The pink and purple nodes below express the genes implicated in modules, while the lines denote node interactions. DEGs: differentially expressed genes, PPI: protein-protein interaction

The common DEGs that were conversely regulated between the two datasets revealed different sets of interactions and networks. Two modules were identified after applying MCODE to the PPI network (Figure 5). Genes MNDA, LILRB2, FPR2, HCK, and FCGR2A clustered by MCODE with the highest MCC rank from CytoHubba were chosen as hub genes.

## Discussion

We identified 66 overlapping DEGs in the two expression profile datasets, of which 44 were conversely regulated. This strategy was chosen assuming that vitamin D may have effects on the immune system that are contrary to those of T1D. The GO analysis results of BP indicated that the conversely expressed intersecting genes were primarily involved in immune-related processes such as cellular response to cytokine stimulus, cytokine-mediated signaling pathway, and positive regulation of response to external stimulus. Furthermore, the overlapping DEGs exhibited enrichment in various KEGG pathways related to the immune system, such as *Staphylococcus aureus *infection, phagocytosis, and neutrophil extracellular trap formation. Insights into the molecular mechanisms of T1D development provided by these enriched processes and pathways could aid in developing novel therapeutic approaches. The MCODE algorithm was employed to detect genes in clusters, and subsequently, the CytoHubba plugin was applied to investigate the PPI network and select hub genes. The CytoHubba plugin offers an easy-to-use interface for investigating significant nodes in biological interactions. It employs 12 different ways to compute node ranking, with the MCC method demonstrating superior performance in the PPI network [21].

The use of bioinformatics research to discover novel diagnostic biomarkers and treatment targets for autoimmune illnesses has grown in recent years. Integrated bioinformatics analysis has been used to identify potential biomarkers for systemic lupus erythematosus, and IFI44 has been identified as a novel biomarker [22]. A recent study has discovered multiple possible diagnostic biomarkers for T1D via bioinformatics analysis [23]. However, our study specifically examined the disruption of gene expression in individuals recently diagnosed with T1D, with a particular focus on vitamin D status. The current study identified five hub genes, MNDA, LILRB2, FPR2, HCK, and FCGR2A, between the two profiling datasets. All these genes are downregulated in T1D but upregulated in vitamin D-treated samples. The reduced expression of these key immune genes likely contributes to the immune dysregulation underlying T1D onset. These hub genes identified in this study might play a crucial role in the development of T1D in vitamin D-deficient patients [17].

Prior research has demonstrated that the MNDA gene can function as a protector for T1D by controlling the activity of the poly (adenosine diphosphate (ADP) ribose) polymerase [24]. The LILRB2 gene belongs to the leukocyte immunoglobulin-like receptor family. It is located on immune cells and interacts with antigen-presenting cells via major histocompatibility complex (MHC) class I molecules. This interaction transmits a negative signal, which prevents the activation of an immune response [25]. Decreased levels of the immune checkpoint LILRB2 remove an essential brake on autoreactive T cells, enabling them to attack and destroy insulin-producing pancreatic beta cells. Separate studies have confirmed FPR2 dysregulation in both T1D patients [26] and during vitamin D supplementation [27]. However, as far as our knowledge extends, no study has investigated the effect of vitamin D on this gene in newly diagnosed T1D patients. The HCK gene was discovered to be elevated in diabetic nephropathy in a bioinformatics study [28]. However, according to our analyzed dataset, HCK is downregulated in recently diagnosed T1D. A meta-analysis study has revealed a significant association between an FCGR2A variant and autoimmune diseases, including T1D [29]. This is because the FCGR2A gene product is a potent immune system modulator that binds autoantibodies and activates immune cells [30].

Overall, the identified hub genes reinforce the immune perturbation at the center of T1D pathogenesis. Their targeted downregulation disables crucial checkpoints that usually keep autoimmune responses in check. Strategies aimed at restoring the expression of these genes, such as vitamin D supplementation, represent a promising approach to reinstating immune homeostasis and averting autoimmune destruction of insulin-secreting cells in genetically prone individuals [17]. Further research must be done to unveil the exact role of vitamin D metabolism on newly diagnosed T1D as well as at-risk subjects such as T1D siblings. The best approach would be an interventional study in vitamin D-deficient T1D followed by whole genome expression profiling of peripheral blood mononuclear cells. It may be helpful to study the role of vitamin D in the development of T1D by including siblings who are deficient in the vitamin and have a higher risk of developing the disease.

This study primarily utilized bioinformatics approaches to identify key immunomodulatory genes associated with T1D and vitamin D metabolism. While these findings provide valuable insights, it is important to note several limitations. Firstly, the study did not include samples from actual T1D patients, which would have yielded more clinically relevant data. The inclusion of such patients could have strengthened the conclusions and increased the applicability of the findings to real-world scenarios. Additionally, the results were not validated through experimental methods such as quantitative real-time polymerase chain reaction (qRT-PCR), which is crucial for confirming gene expression levels. These limitations underscore the necessity for further research to validate the bioinformatics predictions and to investigate the functional significance of the identified genes in T1D patients.

## Conclusions

This study in bioinformatics has successfully identified key immunomodulatory genes that may play a crucial role in the development of T1D, possibly influenced by vitamin D metabolism. By using bioinformatics tools, a comprehensive analysis was conducted, revealing gene interactions that could be targeted in future therapeutic interventions aimed at modulating immune responses in T1D. However, experimental validation is needed to confirm the gene expression levels and their relevance in actual T1D patients. This step is essential to establish whether the identified gene expression patterns are consistently associated with the disease and can be reliably measured in clinical settings. These findings open opportunities for further research to explore the therapeutic potential of targeting these hub genes in the management of T1D.
